# Effect of manufacturing and experimental conditions on the mechanical and surface properties of silicone elastomer scaffolds used in endothelial mechanobiological studies

**DOI:** 10.1186/s12938-017-0380-5

**Published:** 2017-07-14

**Authors:** Marc-Antoine Campeau, Audrey Lortie, Pierrick Tremblay, Marc-Olivier Béliveau, Dominic Dubé, Ève Langelier, Léonie Rouleau

**Affiliations:** 1 Department of Chemical Engineering, McGill University, Montreal, QC H3A 0C5 Canada; 2 Département de génie chimique et biotechnologique, Université de Sherbrooke, Sherbrooke, QC J1K 2R1 Canada; 3 Département de génie mécanique, Université de Sherbrooke, Sherbrooke, QC J1K 2R1 Canada; 4 Centre de recherche du Centre hospitalier universitaire de Sherbrooke, Sherbrooke, QC J1H 5N4 Canada

**Keywords:** Silicone elastomer, Young’s modulus, Viscoelasticity, 3D scaffolds, Cell culture, Endothelial cells, Shear stress, Stretch, Extracellular matrix proteins

## Abstract

**Background:**

Mechanobiological studies allow the characterization of cell response to mechanical stresses. Cells need to be supported by a material with properties similar to the physiological environment. Silicone elastomers have been used to produce various in vitro scaffolds of different geometries for endothelial cell studies given its relevant mechanical, optical and surface properties. However, obtaining defined and repeatable properties is a challenge as depending on the different manufacturing and processing steps, mechanical and surface properties may vary significantly between research groups.

**Methods:**

The impact of different manufacturing and processing methods on the mechanical and surface properties was assessed by measuring the Young’s modulus and the contact angle. Silicone samples were produced using different curing temperatures and processed with different sterilization techniques and hydrophilization conditions.

**Results:**

Different curing temperatures were used to obtain materials of different stiffness with a chosen silicone elastomer, i.e. Sylgard 184^®^. Sterilization by boiling had a tendency to stiffen samples cured at lower temperatures whereas UV and ethanol did not alter the material properties. Hydrophilization using sulphuric acid allowed to decrease surface hydrophobicity, however this effect was lost over time as hydrophobic recovery occurred. Extended contact with water maintained decreased hydrophobicity up to 7 days. Mechanobiological studies require complete cell coverage of the scaffolds used prior to mechanical stresses exposure. Different concentrations of fibronectin and collagen were used to coat the scaffolds and cell seeding density was varied to optimize cell coverage.

**Conclusion:**

This study highlights the potential bias introduced by manufacturing and processing conditions needed in the preparation of scaffolds used in mechanobiological studies involving endothelial cells. As manufacturing, processing and cell culture conditions are known to influence cell adhesion and function, they should be more thoroughly assessed by research groups that perform such mechanobiological studies using silicone.

**Electronic supplementary material:**

The online version of this article (doi:10.1186/s12938-017-0380-5) contains supplementary material, which is available to authorized users.

## Background

Endothelial cells (ECs), lining blood vessels, respond in vivo to mechanical stresses such as wall shear stress from blood flow, axial and circumferential mechanical stretch from movement of blood vessels during cardiac rhythm and hydrostatic pressure imposed radially by blood. Through mechanotransduction, ECs convert mechanical stresses into biochemical signals, altering their morphology, proliferation, apoptosis, migration, differentiation and expression, potentially resulting in the focal development of pathologies such as atherosclerosis [1–3]. Other cell types in the blood vessel wall, such as smooth muscle cells, can also respond to mechanical stimuli.

Mechanical stresses are thus important to consider while performing experiments and should be included during in vitro studies which are conducted to better understand the effect of mechanical forces on cell response. Although in vivo experiments provide a biological and mechanical environment closer to physiological conditions, it is difficult to study the effect of specific mechanical stresses. In vitro mechanobiological studies are thus used to expose cells to controlled mechanical stresses in many different conditions in parallel, allowing higher throughput and reduced cost when compared to in vivo studies. In vitro experiments require scaffolds or experimental cell culture models to grow cells in specific geometries, as well as dynamic bioreactors to impose mechanical stresses. These constructs should be adapted to the cell type studied. In the cardiovascular system, 3D scaffolds can represent geometrically accurate constructs used to seed an endothelial cell EC monolayer but can also represent smooth muscle cells encapsulated into a hydrogel.

Different materials have been used to construct scaffolds for EC mechanobiological studies differing in terms of their mechanical, optical and surface properties. Rigid materials such as glass have been used in the past, including the cone-plate and parallel plate devices [4, 5]. However, compliant materials are nowadays preferred in order to match the geometry and mechanical properties of native arteries. In addition, cell adhesion and proliferation is necessary to form a confluent EC monolayer and mimic blood vessels. Hence, the surface properties of the scaffolds must allow cell adhesion and additionally the bulk material must be suitable for observation of the cells through light microscopy during culture.

Silicone elastomer, polydimethylsiloxane or PDMS is a material of choice in EC mechanobiological studies to produce in vitro scaffolds (membranes, tubes and complex geometries) due to its optimal properties [6–12]. It is manufactured using different molding methods (conventional, injection, dip, dip-spin and sequential molding or more recently additive manufacturing using stereolithography) and often used to test imaging modalities (X-ray, ultrasound, magnetic resonance) and medical devices (stents, valves) as well as in microfluidic devices [6, 13–17]. Silicone is biocompatible and nontoxic [18]. It is permeable to gases, allowing gas exchange with the surroundings and ensuring controlled pH through buffering of the cell culture media. As it is hydrophobic, its surface needs to be hydrophilized to allow extracellular matrix protein coating and subsequent cell adhesion and proliferation. Different procedures have been used to alter silicone surfaces, such as oxidization by exposure to plasma and etching using strong acids [19]. Silicone has excellent optical properties, as it is transparent, non auto-fluorescent and has an easy to match refractive index. Sylgard 184^®^ is a commercial (Dow Corning), relatively low cost, two part heat curable silicone elastomer [16, 17]. In the context of mechanobiological studies, it has adequate mechanical properties being isotropic and displaying a nearly linear Young’s modulus in the strain range considered for physiological testing (slightly viscoelastic). It has been used to produce both rigid and compliant scaffolds to study the effect of wall shear stress [10, 11, 17] and mechanical stretch [20, 21] on endothelial cell function. It is possible to modify the rigidity of the material by changing curing parameters and it can also be mixed with additives such as Xiameter PMX-200 [12] or with Sylgard 527^®^ [22].

Different parameters altering cell adhesion and function have been previously characterized. Cell adhesion, spreading and proliferation can be altered by substrate stiffness. Evans et al. [23] reported that proliferation and spreading of embryonic stem cells cultured on silicone was increased on stiffer matrices particularly at longer time points. Similar results have been found by other research groups for fibroblasts (NIH 3T3), endothelial cells and vascular smooth muscle cells, suggesting that cell spreading is increased by stiff substrates [24–26]. It has also been reported that expression of focal adhesion kinase differ with varying matrix stiffness, thus influencing cell spreading and proliferation but also gene expression and cell function [23]. Surface hydrophobicity has been found to affect substrate adhesion and conformation and hence, cell adhesion. Silicone surface can be modified by plasma treatment, thus altering hydrophobicity, decreasing contact angle and thus enhancing the effective binding of extracellular matrix proteins such as fibronectin to the surface [27, 28]. Increased adhesion of fibroblasts to silicone surfaces treated with plasma has been observed [29]. Alternatively and at a lower cost than plasma treatment, silicone can be hydrophilized by strong acids [11]. Hydrophilization treatment and fibronectin coating may have a synergic effect on cell adhesion as shown by Colombo et al. [30].

Different studies have demonstrated that the density of cell–matrix adhesion ligands influences cell behavior. Higher concentrations of adsorbed extracellular matrix molecules have been shown to increase cell adhesion, spreading and proliferation [31–33]. In addition to density and concentration of substrates, the conformation of the extracellular matrix molecules has also been shown to influence cell behavior. For instance, different coating techniques resulting in different collagen conformations due to hydrophobic interactions, alter cell response as suggested by Wipff et al. [28].

In this manuscript, results for Sylgard 184^®^ are presented as it is the material most often used to grow endothelial cells in 3D geometries for mechanobiological studies. The manufacturing and molding techniques of silicone scaffolds differ between research groups but consist essentially in degassing, using vacuum pumps [10, 11] or centrifuges, followed by curing at variable temperatures, using ovens with natural or forced convection. Air removal is essential as light would be refracted from trapped air bubbles, thus preventing adequate observation through microscopy, or as anisotropy in the material would cause localized stress variations, and potential differences in cell response. Curing parameters (time and temperature) as well as the base to curing agent ratio are commonly used to produce scaffolds with different stiffness, allowing the production of physiologically relevant scaffolds [28, 30]. As detailed above, these stiffness changes can result in difference in cell response and thus it is important to characterize changes in mechanical properties due to processing techniques.

The comparison of EC mechanobiological studies from different research groups is still challenging as the impact of the different manufacturing and processing parameters influencing the mechanical and surface properties are not yet fully characterized, thus resulting in potential differences between research groups. In this study, we have used Sylgard 184^®^, molded as membranes and as tubular compliant in vitro scaffolds, to characterize the effect of these parameters on the mechanical and surface properties of the material. The viscoelasticity of the material was determined through measures of hysteresis at different strain rates. Influence of the curing temperature and sterilization technique was assessed through mechanical tests, by extracting engineering stress–strain curves and calculating the Young’s modulus for five evenly distributed strain ranges. Effect of the hydrophilization treatment to reduce surface hydrophobicity was quantified through measure of the contact angle. Hydrophobic recovery was studied upon different storage conditions, up to 7 days in water or alternatively in air. Optimal cell culture conditions were assessed by varying the type and concentration of the substrate coating as well as the cell seeding concentration in tubular compliant scaffolds.

## Methods

### Manufacturing conditions of silicone membranes

Silicone elastomer (Sylgard 184^®^, Dow Corning, distributed by EBPeerless, Canada), was prepared according to the instructions of the manufacturer. The base and curing agent were combined at a 10:1 volumetric ratio or 9.09% v/v as measured using syringes. It was thoroughly mixed and degassed using a vacuum pump and chamber assembly for 30 min. The mix was transferred using a syringe (for a constant volume) in an aluminum mold, ensuring rapid and uniform heat conduction. Remaining bubbles in the mix were allowed to rise and escape as the mold was left on the counter for 30 min. The silicone was cured for 4 h in a controlled gas chromatography forced convection oven, allowing rapid change and control of the temperature profile during rise and descent. A curing period of 4 h was arbitrarily chosen for the ease of production, i.e. the possibility to perform the entire sample manufacturing process over a usual workday. Curing temperatures of 50, 80, 100 and 150 °C were used. Molds for manufacturing the membranes (used for mechanical tests) and the in vitro scaffolds (used for optimization of cell culture) were made of the same material and with the same relative thickness, ensuring similar heat transfer profiles.

### Characterization of mechanical properties of silicone using membranes

**Fig. 1:**
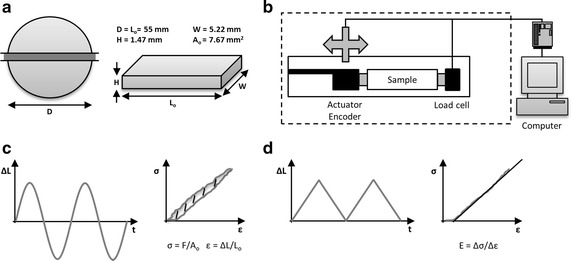
Mechanical tests scheme. **a** Cutting of the samples for tensile tests from membranes with resulting dimensions shown in the figure. **b** Insertion of the samples in the bioreactor and equilibration to 37 °C prior to testing. Displacement imposed using an actuator and encoder assembly and force measured using a load cell. **c** Measurement of the viscoelasticity performed by cyclic sinusoidal testing and measurement of the hysteresis, the energy stored in the material, by computing the area between the loading and unloading curve using Matlab. **d** Experimental data obtained from linear tests and engineering stress and strain was computed. Plotting of the resulting stress-strain data was performed and the Young’s modulus obtained

Circular silicone membranes of 5.5 cm diameter were produced and used for mechanical characterization (Fig. 1a). The height of the membranes was calculated based on the volume transferred and the dimensions of the mold and was verified using a digital caliper prior to mechanical testing. Rectangular samples were cut from the membranes using a custom cutter, resulting in samples with these dimensions: 55 mm length, 1.47 mm height and 5.22 mm width. The initial cross-sectional area of the sample for tensile tests was thus 7.67 mm^2^ (Fig. 1a). A bioreactor [34–36] was used to perform traction tests (Fig. 1b). An anchoring system was specially designed to allow the fixation of the silicone test samples in the bioreactor. Samples were positioned and squeezed between metal plates held in place using a set of two tightened screws. If apparent slipping occurred, the test was redone. The samples were allowed to reach the temperature of standard mechanobiological experiments in an incubator (i.e. 37 °C). The samples were installed in the bioreactor and the point of zero strain was achieved by applying a force of 100 g (0.133 N). The distance between the two anchors of the sample was measured optically by the bioreactor (as described previously) [35].

Two different sets of experiments were carried out, one imposing cyclic sinusoidal testing and another using ramps, all under displacement control (0–28%). Data of force and displacement were recorded as a function of time using Labview (National Instruments) and analyzed using custom Matlab codes, transforming displacement (mm) into engineering strain (%) (dividing by initial length) and force (grams) into engineering stress (MPa) (dividing by initial cross-sectional area). The data was truncated to separate the different cycles using Matlab and the engineering stress–strain curves were obtained as well as the incremental Young’s modulus. Data was then transferred to Excel (Microsoft) and analyzed in Prism 5 (GraphPad). In all experiments, the samples were not brought to rupture and no tear was observed at the anchoring points.

Sinus waveforms, for a total of 13 cycles, were imposed at different frequencies (0.5, 1, 2 Hz) and a displacement amplitude of 28% (Fig. 1c). The hysteresis as defined by the area between the loading and unloading curve was determined in order to characterize the viscoelasticity of the material. The mechanical properties of the silicone elastomer were determined using stretch and release cycles (13) at different strain rates (0.5, 5 and 10%/s) normalized to the sample initial length. The Young’s modulus was computed, compiled, compared and analyzed for the whole engineering strain range of 0–28%, as well as for 5 evenly distributed ranges (0.0–5.6, 5.6–11.2, 11.2–16.8, 16.8–22.4, 22.4–28.0%).

### Sterilization techniques

Scaffolds need to be sterilized prior to substrate coating and cell seeding, thus ensuring adequate cell culture conditions. Different methods, used by different research groups, were tested to sterilize the silicone, i.e. ultraviolet light exposure for 30 min (UV, inside a biosafety cabinet at approximately 50 cm from light source), immersion in ethanol 70% (subsequent drying at room temperature) and boiling in deionized water for 30 min. Membranes were produced, sterilized with these different treatments and their mechanical properties were quantified (using the stress–strain curves and the incremental Young’s modulus). Samples were also produced and used to assess the impact of sterilization on surface properties through goniometry, as described below.

### Hydrophilization using sulphuric acid

Sulphuric acid (Fisher, A300) 70% was prepared in deionized water to hydrophilize silicone surfaces, a process used to enhance substrate and cell adhesion. Membranes were immersed in the sulphuric acid solution at room temperature for different durations (15, 30 and 60 min) and the contact angle was measured to verify the effect of hydrophilization immersion duration.

### Storage conditions

As samples are sometimes stored prior to experiments, silicone membranes were stored in distilled water or in air at room temperature, in six well plates for durations up to 7 days following hydrophilization and surface contact angle measurements.

### Contact angle measurements through goniometry

As changes in the wettability of the surface can influence the conformation of the proteins to be adsorbed along with the cellular response associated, contact angle measurements were performed. A goniometer (OCA10, Data physics, SCA20 software) was used to assess the surface properties of the silicone. Prior to contact angle measurements, samples were rinsed four times with deionized water. Membrane samples were laid flat on a glass slides and installed in the apparatus. Deionized water drops of 3 µL were deposited on the samples using the goniometer electronic control syringe module, using a 500 µL automatic push syringe. The contact angle of sessile drops deposited on the silicone surface was measured through the SCA20 software. Manual identification of the silicone surface allowed the software to detect the limits of the liquid drop and the contact angles were measured for each side of the drop. The data was typed in Excel (Microsoft) and transferred to Prism 5 (GraphPad) to be analyzed.

Membranes cured at different temperatures (50, 80, 100 and 150 °C), sterilized using the different techniques, immersed for different durations in sulphuric acid (15, 30 or 60 min) and stored in different conditions and durations were tested.

### Production of compliant tubular in vitro scaffolds for cell culture experiments

**Fig. 2:**
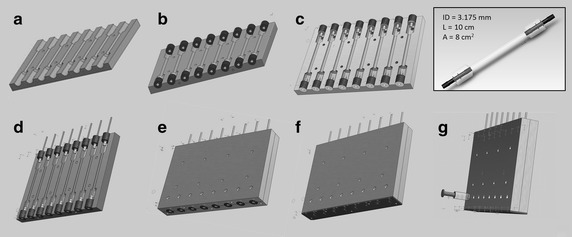
Mold assembly. **a** Half of the mold. **b** Installation of the rubber stoppers. **c** Insertion of the connectors halfway in the stoppers. **d** Insertion of the rods through the assembly. **e** Installation of the other half of the mold. **f** Installation of the base (*bottom*). **g** Assembly of the mold using screws. Filling of the mold from the top by putting the connector/rubber stopper assembly above the mold and pouring the degassed silicone mix. Replacement of the connector/rubber stopper assembly and resting for 30 min. Resulting dimensions of the tubular scaffold are shown in figure

Compliant tubular scaffolds were produced with a custom designed mold (Fig. 2) using silicone prepared as described previously and a curing temperature of 100 °C. Stainless steel rods (3.175 mm inner diameter, 1256T12, McMaster Carr) and connectors (06365-22, 30622-57, Cole Parmer) were used to form the lumen of the constructs at the center of the mold. Rubber stoppers were used to cover part of the connectors allowing alignment of the lumen within the construct while preventing silicone to cover that area. After curing, the rods were removed to create compliant tubular in vitro scaffolds. To pull out the inner rod, air was allowed to enter between the silicone and the rod for easier movement of the rod while keeping it parallel to the surface, thus preventing the formation of surface defects. Scaffolds were hydrophilized for 45 min using the method described above. Following hydrophilization, surfaces were rinsed thoroughly with tap water, scaffolds were sterilized in boiling deionized water for 30 min and were allowed to cool prior to substrate coating and cell seeding.

### Substrate coating in scaffolds

**Fig. 3:**
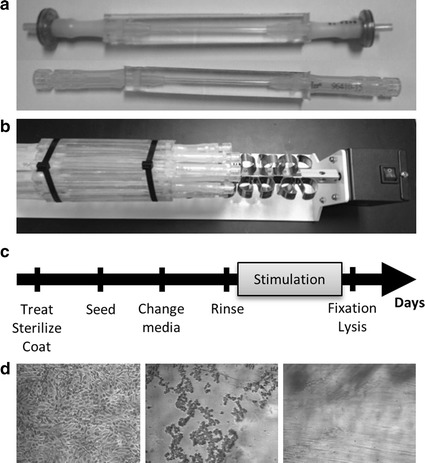
Cell culture considerations. **a** Experimental set-up. Tubing = filter or tubing plug. **b** Arrangement for even coverage. **c** Overall cell culture process. **d** Efficient trypsinization of cells is assessed using microscopy

To obtain a uniform endothelial cell monolayer within the scaffolds, it is important to determine the substrate coating type and concentration that will lead to optimal cell coverage. The tubular scaffolds were prepared as described and their lumen coated with different concentrations of collagen type 1 (5–10 µg/mL, C3867, Sigma-Aldrich) and/or fibronectin (10–40 µg/mL, F0895, Sigma-Aldrich) in 1× Phosphate buffer solution (PBS). The solutions were pipetted in the lumen of the scaffolds and filters were inserted with tubing at both extremities (Fig. 3a). Alternatively, a plug system can be used to enhance sterility while samples are manipulated. Substrate coating was performed overnight at 37 °C while the scaffolds were attached on a rotor (8 rpm) (Labquake Rotor, Series 1104, Barnstead/Thermolyne) (Fig. 3b). Coating solutions were pipetted out of the scaffolds and the scaffolds were washed once by pipetting 1× PBS in and out of the lumen before cell seeding. This ensured the removal of unbound substrate that could prevent cells to adhere to the surface.

### Cell seeding in tubular scaffolds

Cell seeding concentration is another key parameter to assess and optimize to obtain a uniform cell monolayer. Human abdominal aortic endothelial cells (Line HAAE-1) derived from a 20-year-old male were purchased from the Coriell Institute for Medical Research (AG09799) and expanded up to passage 5. The cells were grown on gelatin-coated tissue culture flasks (0.1%, G1890, Sigma) with endothelial cell growth medium (C-22010, C-39215, Promocell), supplemented with 10% fetal bovine serum (26140-079, Gibco, Invitrogen), and 1% penicillin streptomycin (15140-122, Gibco, Invitrogen). At confluency, cultures were rinsed with 1× PBS, harvested with 0.25% Trypsin–EDTA (25200-072, Invitrogen) gathered in 15 mL centrifuge tubes, centrifuged at 1200 rpm for 5 min and resuspended at different concentrations (2.5 × 10^5^, 5.0 × 10^5^ and 10.0 × 10^5^ cells/mL). The lumen of the tubular scaffolds had an inner diameter of 3.175 mm, thus the seeded cell volume for a tube length of 1 cm was 0.08 mL, whereas the area for culture in that volume was about 1 cm^2^.

The cell seeding solution was pipetted in the lumen of the scaffolds and cells were let to adhere for 2 min on the surface of the scaffold. The tube was then turned horizontally by 90°, and let to adhere for 2 min, and this process was repeated until all sides had been in contact with the cells. Tubes were then attached on the rotor (8 rpm) allowing even coverage of ECs on the surface and incubated at 37 °C, 5% CO_2_. Growth medium was changed the day following seeding (24 h post seeding). Cells were incubated for another 24 h, imaged using light and fluorescence microscopy, trypsinized out of the scaffolds (48 h post seeding) and counted. The procedure including stimulation for a defined period is shown in Fig. 3c.

### Assessment of cells adhered in tubular scaffolds

Effect of substrate type and concentration as well as cell seeding concentration was assessed based on the number of cells present in the tubular scaffolds after 48 h of growth. Live cells were stained with Hoescht 33342, a nucleic acid cell-permeant nuclear counterstain (dsDNA) emitting blue fluorescence (10 mg/mL, used at 1:10 000 or 1 μg/mL for 30 min, H-3570 Invitrogen). They were washed three times with 1× PBS and imaged in situ on a Nikon Eclipse TE-2000U microscope using appropriate filters and light source. Some scaffolds were stained with crystal violet 4% in 1× PBS (212525, BD Biosciences) and imaged using light microscopy.

After fluorescent staining and imaging, cells were rinsed with 1× PBS and trypsinized out of the lumen. Trypsinization was performed by inserting trypsin in the lumen of the scaffolds (0.8 mL). The scaffolds were incubated for 5 min at 37 **°**C and cells were observed at the microscope to verify cell detachment from the surface (Fig. 3d). Gentle mechanical pressure was applied on the length of the scaffolds to ensure even removal of the cells from the surface. Cells were gathered in tubes (2 mL) and centrifuged at 12,000 rpm for 5 min. Cells were resuspended in 100 μL of 1× PBS and counterstained with trypan blue (100 μL, 0.4% solution, 15250061, Fisher) to assess the number of cells recovered using an hemocytometer.

### Statistical analysis

Results are expressed as mean ± standard deviation. The number of experiments performed is written in each caption. Prism 5 (GraphPad) was used to analyze the results. Mean values were compared using two-way analysis of variance (ANOVA) followed by Bonferroni post-tests with a 95% confidence interval. P-values less than 0.05 were considered statistically significant (p < 0.05 *, p < 0.01 **, p < 0.001 ***).

## Results

### Effect on mechanical test conditions on stress–strain curves and viscoelasticity

**Fig. 4:**
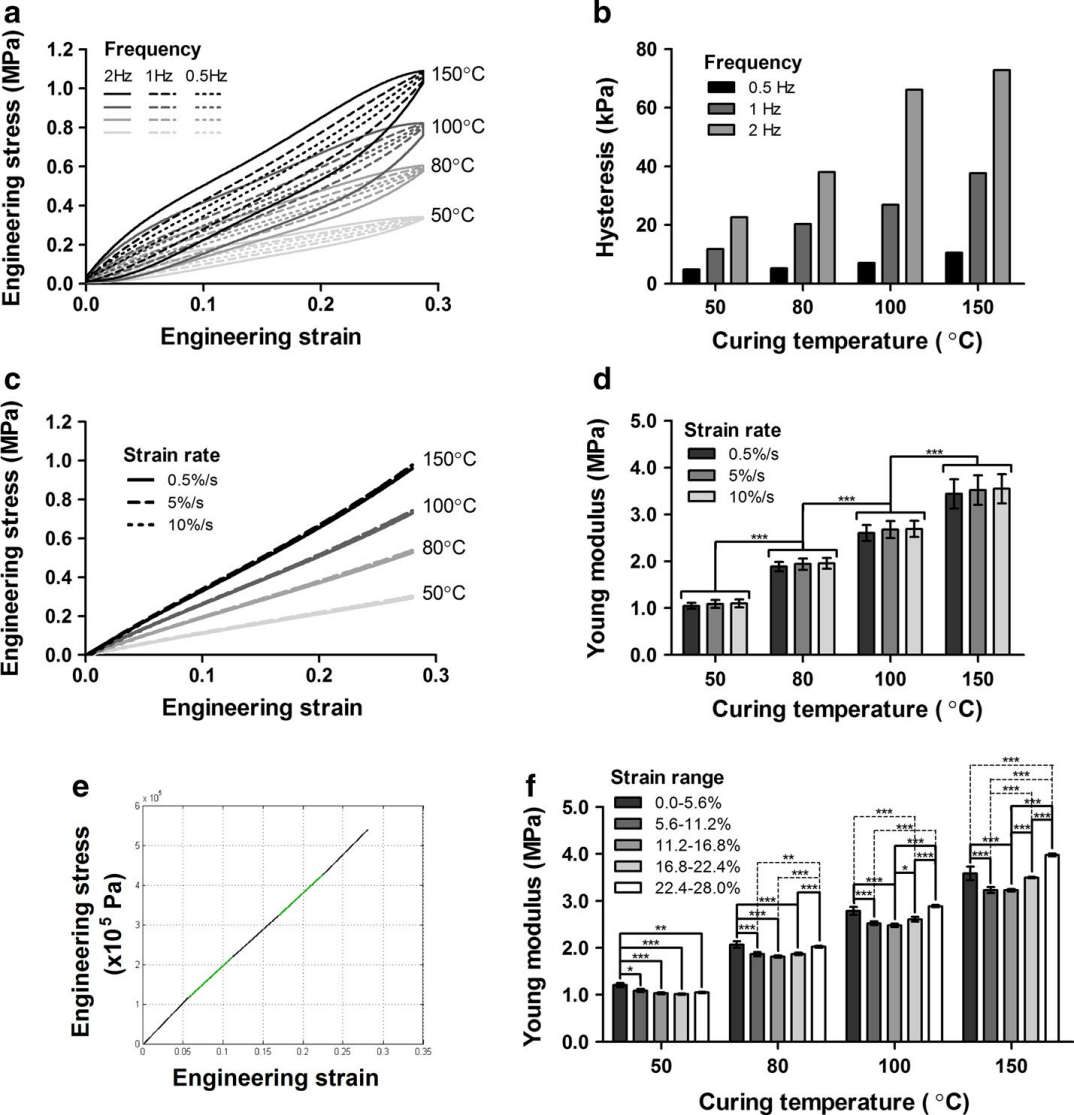
Effect of frequency (0.5, 1, 2 Hz) on stress–strain curves (**a**) and on hysteresis (**b**). Effect of strain rate (0.5, 5, 10%) on stress–strain curves (**c**) (loading curve shown) (n = 1) and on Young’s modulus (**d**) for different curing temperatures (n = 5) (Two-way ANOVA, significant effect of curing temperature (***p < 0.0001), significant differences between temperatures, no differences between strain rates). Stress–strain curve highlighting the five different strain ranges considered (**e**) (n = 1, 80 °C). Effect of strain range on Young’s modulus (**f**) for different curing temperatures, strain rates averaged (n = 3) (Two-way ANOVA, significant effect of strain range (***p < 0.0001) and curing temperature (***p < 0.0001), significant differences between all temperatures)

As silicone is a viscoelastic material, mechanical cyclic sinusoidal tests have been performed to characterize the material. As it is to be used in physiologic conditions, it was tested at 37 °C and frequencies between 0.5 and 2 Hz at an engineering strain of 28%. Samples cured at different temperatures were tested as seen in Fig. 4a. The loading and unloading curves for these sinusoidal tests were different and varied as a function of both curing temperature and frequency. The slowest frequency resulted in curves closer to each other compared to rapid cycling. The hysteresis was greater at higher frequency and higher curing temperatures (Fig. 4b).

Stretch and release cycles were performed at varying strain rates to examine the potential effect on the engineering stress–strain curves (Fig. 4c). At the strain rates considered, the loading and unloading curves were very similar, hence only loading curves are shown. The effect of strain rate was quantified by computing the Young’s modulus for the overall engineering strain range (0–28%) (Fig. 4d). The Young’s modulus was not found to be different between the strain rates for a given curing temperature as determined using a two-way ANOVA and multiple comparisons post-test.

To further examine the viscoelastic behavior of silicone, the effect of the strain range considered was analyzed. The 0–28% range of the tests performed was divided in five sections as shown in Fig. 4e. Curves appeared to have one or more inflection points due to apparent changes in their slope, with steeper curve regions at lower and higher strains. According to our data, silicone has demonstrated a slightly viscoelastic behavior in the range considered. The incremental Young’s modulus was found for the different ranges of the engineering stress–strain curves and analyzed, as shown in Fig. 4f. As found by a two-way ANOVA, the data revealed a significant effect of the temperature but also a significant effect of the strain ranges on the Young’s modulus.

### Effect of curing temperature and sterilization technique on mechanical properties

**Fig. 5:**
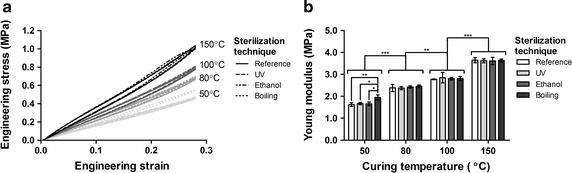
Effect of sterilization technique (UV, ethanol, boiling) on engineering stress–strain curves (**a**) (n = 3) and on the Young’s modulus (**b**) for different curing temperatures (n = 3) (Two-way ANOVA, significant effect of curing temperature (***p < 0.0001), significant differences between all temperatures, significant differences between sterilization techniques at 50 °C only)

Engineering stress–strain results demonstrate that higher temperatures of curing stiffen the material as quantified using the Young’s modulus (Fig. 5a). The Young’s modulus increased with higher temperatures in the range tested (50–150 °C) and significant differences were found (Fig. 5b), with values between 1.7 and 3.7 MPa.

Samples cured at different temperatures and sterilized by UV, ethanol or boiling water were tested and compared to reference samples of their corresponding curing temperature (manufactured at the same time, unexposed to UV, ethanol or boiling water). The engineering stress–strain curves showed that boiling had a significant effect on the shape of the curves, especially at lower temperatures of curing (50 °C), whereas the other sterilization techniques did not (Fig. 5a). The Young’s modulus was also affected by boiling for the lowest curing temperature (50 °C), whereas at higher temperatures of curing the effect is not present (Fig. 5b). Hence, heat related sterilization techniques alter the mechanical properties of the material.

### Effect of curing temperature and immersion duration on contact angle

**Fig. 6:**
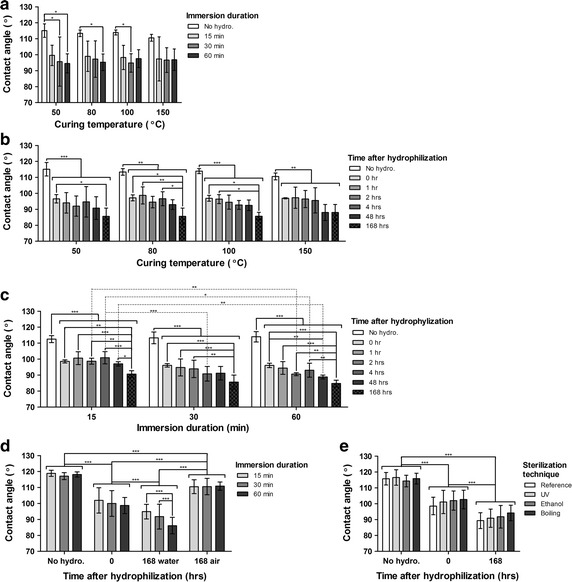
Effect of hydrophilization and immersion duration (15, 30, 60 min) on contact angle (**a**) for different curing temperatures (n = 3) (Two-way ANOVA, significant effect of immersion duration (***p < 0.0001), significant differences between no immersion (No hydro.) and different immersion durations). Effect of hydrophilization and time after hydrophilization (No hydro, 0, 1, 2, 4, 48, 168 h) on contact angle for different curing temperatures (**b**), immersion duration triplicatas averaged (n = 3). (Two-way ANOVA, significant effect of time after hydrophilization (***p < 0.0001), significant differences between No hydro. and all other timepoints, significant differences between timepoints after hydrophilization). Effect of hydrophilization and time after hydrophilization (No hydro, 0, 1, 2, 4, 48, 168 h) on contact angle for different immersion durations (**c**), curing temperature triplicatas averaged (n = 4). (Two-way ANOVA, significant effect of time after hydrophilization (***p < 0.0001), significant differences between No hydro. and all other timepoints, significant differences between timepoints after hydrophilization, significant effect of immersion duration (***p < 0.0001), significant differences between immersion durations). Effect of storage time and conditions (No hydro., 0, 168 water, 168 air) on contact angle for different immersion durations (**d**), curing temperature quadruplicatas pooled (n = 32). (Two-way ANOVA, significant effect of immersion duration (***p < 0.0001), significant differences between immersion durations for 168 h in water, significant effect of time after hydrophilization (***p < 0.0001), significant differences between all timepoints and conditions). Effect of sterilization technique (UV, ethanol, boiling) on contact angle for different times after hydrophilization (**e**), curing temperature triplicatas pooled (n = 12). (Two-way ANOVA, no effect of sterilization technique, significant effect of time after hydrophilization (***p < 0.0001), significant differences between all timepoints)

Silicone membranes were tested prior to hydrophilization (No hydro.) to determine the effect of the curing temperature on the contact angle. No effect of the curing temperature on the contact angle was found using a two-way ANOVA analysis (Fig. 6a). The samples cured at different curing temperatures were then immersed in sulphuric acid for different durations (15, 30, 60 min) and the contact angle was measured after rinsing in tap water four times. Significant differences were found between reference samples (No hydro.) and those immersed for different durations in sulphuric acid. However, no difference between immersion durations (15, 30, 60 min) was found using a two-way ANOVA analysis, indicating that the extent of change in contact angle is attained after 15 min. Furthermore, it has been verified that hydrophilization by immersion in sulphuric acid for up to 60 min has no effect on silicone stiffness (data not shown).

### Effect of storage time and conditions on contact angle

The effect of storage time, i.e. time after hydrophilization, was observed for samples kept in water or air for durations up to 7 days (168 h). Contact angle varied in function of time after hydrophilization for the different curing temperatures (Fig. 6b) and sulphuric acid immersion durations (Fig. 6c). Data for different immersion durations and curing temperatures were averaged respectively. Storage in water significantly altered the contact angle. Lower contact angles were found for samples exposed to water for an extended duration (Fig. 6b, c). No significant effect of the curing temperature was found (Fig. 6b). Interestingly, while the immersion duration in sulphuric acid seemed to have no effect on the contact angle right after hydrophilization (Fig. 6a), such an effect was found following storage in water using a two-way ANOVA analysis. Hence, longer immersion in sulphuric acid seemed to enhance the effect of storage in water resulting in lower contact angles at longer storage times (2, 4, 48 h) (Fig. 6c).

As previously noted, an effect of time after hydrophilization was found and all conditions resulted in significantly different contact angle measurements (Fig. 6d). However, samples kept in air tended to return to their original contact angle, an effect known as hydrophobic recovery, whereas samples kept in water did not. Furthermore, an effect of the immersion duration was found for samples kept in water for 168 h, with decreasing contact angles for increasing immersion duration, supporting the trend found in Fig. 6c.

Contact angle for samples sterilized using the different techniques (UV, ethanol and boiling) and hydrophilized for 45 min (data of curing temperatures averaged) was also measured. Sterilization techniques had no effect on this parameter (Fig. 6e). Also, the storage conditions (water or air) has been shown to have no effect on the stiffness of the material after a storage period of 7 days. However, an aging effect, i.e. a gradual stiffening of the material stored, was observed as an increased Young’s modulus was found for sample kept in water and air for 7 days (see Additional file 1).

### Effect of substrate concentration and type on cells in the scaffolds

**Fig. 7:**
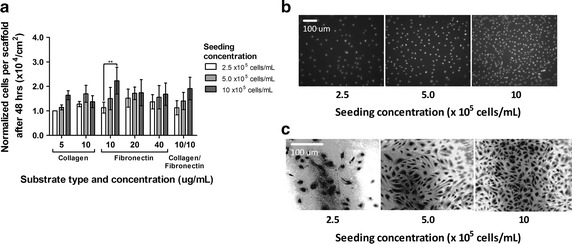
Effect of concentration of cell seeded on number of cells recovered from cell culture scaffolds for different substrate type and concentration (**a**), data normalized to Collagen 5 µg/mL, 2.5 × 10^5^ cells/mL (n = 3). Two-way ANOVA, significant effect of concentration of cell seeded (***p = 0.0003), significant difference between concentration seeded for 10 µg/mL fibronectin. Assessment of cell coverage through fluorescent nucleus staining (**b**), Hoescht 33342, 1 μg/mL, 30 min (bar length = 100 µm, magnification = 40×, Nikon Eclipse 2000 microscope) and through crystal violet staining (**c**) (bar length = 100 µm, magnification = 40×) for different concentrations of cell seeded

Different substrates can be used to enhance cell adhesion to silicone. Collagen and fibronectin were tested alone and in combination in the tubular scaffolds. No significant effect of substrate was found using a two-way ANOVA. Based on cells gathered after 48 h of culture, using collagen alone, or in combination with fibronectin, is equivalent to using fibronectin alone. Increasing substrate concentration did not increase cell adhesion (Fig. 7a).

### Effect of cell seeding concentration

Cell coverage of the tubular scaffolds was assessed visually using both a Hoescht and a crystal violet stain. The effect of cell seeding concentration was quantified by gathering cells from the scaffolds. A significant effect of the seeding concentration on the number of cells gathered from the scaffolds after 48 h was found using a two-way ANOVA (Fig. 7a) but significant difference was reached for only one coating condition. Nevertheless, a trend is depicted for most coating conditions, showing that increasing cell seeding concentration may increase cell adhesion. This trend was confirmed by direct observation of the cells in the tubular scaffolds through staining and the formation of an endothelial cell monolayer was clearer with increasing seeding concentration (Fig. 7b, c). A minimal cell seeding concentration of 10 × 10^5^ cell/mL (density of 8.0 × 10^4^ cells/cm^2^) appeared to be required to form a monolayer after 48 h of culture in the scaffolds from the microscope images obtained.

## Discussion

As ECs sense and respond to mechanical stresses, they have been extensively studied in mechanobiological studies to elucidate their role in the development of cardiovascular diseases such as atherosclerosis. In the past, cone-plate and parallel plate in vitro scaffolds were used to conduct such studies. In the last decade, realistic in vitro scaffolds were produced to provide a more realistic culture environment for ECs by being representative of blood vessel geometry (tubular) and thus the use of these scaffolds represent an asset when it comes to mechanobiological studies. Silicone elastomer is used to create tubular scaffolds as well as more complex geometries. The manufacturing techniques, the different steps required to render silicone hydrophilic and allow cell culture, as well as the experimental conditions used in mechanobiological studies differ greatly between research groups and the characterization of the impacts of the techniques and conditions used currently lacks in the literature. In this context, we have tested and examined a broad range of relevant parameters to highlight the potential biases induced by differences in mechanical and surface properties of the silicone elastomer.

### Manufacturing

The technique used in this study to manufacture the tubular scaffolds is efficient and reproducible, allows the production of membranes and compliant tubular scaffolds. The removal of air bubbles trapped in the mix was ensured in our case by using a vacuum pump and chamber assembly, however, other groups have used planetary centrifugal mixers [17], thus combining high speed revolution and rotation. Incomplete degassing is particularly important when considering thin compliant scaffolds as defects could cause anisotropy in the material properties and regional differences in stresses imposed. The procedures used are however rarely detailed in manuscripts and should be better characterized.

Sylgard 184^®^ is a silicone elastomer that requires heat to cure. Standard laboratory ovens with natural convection can be used as well as controllable forced convection ovens, altering the temperature patterns experienced by the material and potentially resulting in differences in mechanical properties. In this study, we have used a gas chromatograph forced convection oven allowing both heating and cooling of the mold containing the silicone. Natural convection ovens can also be used but it seemed that the heat transfer was not as controlled, especially for higher temperature, thus resulting in altered stiffness (data not shown).

The geometry and the material of the mold is also important to consider as these parameters alter the heat transfer to the silicone. This has rarely been described in published protocols and thus it is difficult to compare the mechanical properties obtained with similar molding techniques and mixing ratios, when the mold is unknown. This could alter significantly the results in terms of mechanical properties and consequently cell adhesion and expression.

### Mechanical properties

According to the manufacturer, the mechanical properties of silicone elastomer prepared with the standard base to curing agent ratio (10:1) has a range of Young’s modulus between 1 and 2.5 MPa. Although it is possible to alter the mechanical properties by varying the base to curing agent ratio, we have chosen to use different curing temperatures and a constant curing time with a fixed base to curing agent ratio of 10:1. We have obtained Young’s modulus values ranging from 1.00 to 4.00 MPa for curing temperatures of 50 and 150 °C respectively. The production of materials with different rigidities has allowed us to compare the alteration of the post-processing techniques, such as hydrophilization and sterilization on the mechanical properties.

Changes in the base to curing agent ratio have been studied extensively. The more the PDMS is crosslinked, the less it is elastic [16]. We have decided to use the recommended ratio, as it is the most commonly used, and should result in no crosslinking agent left in the silicone elastomer, which could potentially leach out and affect cells [23, 24, 26, 37, 38]. As expected, adding more base than the recommended amount results in softer material, however, increasing the amount of curing agent does not significantly change the properties [30, 39].

Similar to our results, Liu. et al. have examined as well the effect of curing temperature and time on the mechanical properties of silicone, using a high temperature range (100, 150, 200, 300 °C) and varying times (between 30 min and 3 h). The material was stiffer at longer times and higher temperatures except at temperature above thermal decomposition (>200 °C) [18]. Schneider et al. have found a linear elastic modulus of 1.76 MPa for a curing temperature of 150 °C (curing time of 15 min), in a strain range up to 45% [40], while Fuard et al. used a curing temperature of 100 °C and found that the modulus could vary between 0.75 and 2 MPa if curing time increased from 15 min to 3 h [29]. These variations in the resulting mechanical properties for similar curing (temperature and time) conditions highlight the importance of having a reliable method to produce the scaffolds and to detail the manufacture technique of the scaffolds in the manuscripts as slight differences in curing temperature and time can alter stiffness of the resulting material.

As hydrophilization is a surface treatment, it is not expected to affect the material bulk properties. Strong acids modify chemical bonds at the surface of the silicone, which doubtfully contribute to the material stiffness. We have tested the effect of hydrophilization on the mechanical properties of the silicone and as expected, this surface modification process did not alter the bulk material stiffness (data not shown). Similarly, we are confident that coating would not induce change in mechanical properties of the silicone.

Several methods are available to sterilize scaffolds (UV exposure, ethanol immersion, boiling or autoclaving). We have tested them (except autoclaving), to examine their potential impact on the stiffness of the silicone produced at different temperature. Autoclaving can change the aspect of the material due to swelling, rendering the material opaque, thus losing transparency, which is not desired for imaging purposes and during the seeding procedure (data not shown). As expected, we have found that boiling can affect the mechanical properties of the silicone, by increasing the stiffness of the material. We have found a 20% increase in the Young’s modulus due to boiling for samples cured at 50 °C, as opposed to no difference for materials cured at higher temperatures. Hence, softer materials will be more affected by a thermal related sterilization technique when compared to stiffer materials. Temperature-related processes can further alter the properties of the silicone once it has cured. We have found an increase in Young’s modulus with boiling. This is in agreement with Mata et al. that have found an increase in Young’s modulus with autoclave sterilization [19], and identified no difference between untreated samples, UV or ethanol sterilization techniques. Interestingly and similar to us, they have found statistically significant increase in Young’s modulus with softer samples. Although we have not tested autoclave sterilization, we believe we would have obtained an increase in rigidity as thermal-related sterilization can impact silicone cured at temperature below the one used for sterilization by further contributing to the curing of the silicone to some extent.

Stability of the mechanical properties over time has rarely been studied, although it is important as scaffolds will be produced in batch, kept and used later on. Fuard et al. [29] have examined the evolution in Young’s modulus for silicones of varying base to curing agent ratios and found a reduced stability for the softer materials as studied along a 5 month period. Similarly, Eddington et al. [41] have found that thermal aging for periods up to 14 days can alter the surface properties of silicone. Similarly to these studies, we have found that the mechanical properties of silicone can be altered in time on the order of weeks (see Additional file 1).

### Experimental mechanical testing conditions

There is a lack of standard when it comes to testing mechanical properties of silicone, as different experimental set-ups, mainly compression/indentation testers, dead-weight systems, bioreactors or Instron machines and test sequences (maximum stretch, cycles, strain rate) are used by different research groups. Compression/indentation tests are generally performed in microelectromechanical applications on small samples [23, 42]. Dead-weight [19] and tensile testers (Instron, Bose, Zwick) have been used to assess the properties of rectangular strips [18, 19, 40, 42–44] and dogbone samples [17]. Differences in the characterization method and sample size used can explain to some level the discrepancy between experimental mechanical properties values found. It is thus important for each research group to explicitly mention the technique used to quantify the mechanical properties of their samples as well as their testing procedure.

The strain range is important to consider as silicone is a viscoelastic material behaving linearly up to strain of approximately 75–100% depending on the curing conditions (base to curing agent ratio, time, temperature) used [40, 43]. Endothelial cell mechanobiological studies aim to mimic blood vessels and thus the mechanical properties should match native artery behaviors, especially at low and physiologically relevant strains [30]. While strains imposed on blood vessels vary in the different regions of the vasculature, they exhibit slightly non-linear mechanical properties which become rapidly more apparent at higher strain ranges used mainly in ex vivo mechanical tests [45]. However, in the low strain region considered relevant for reproducing in vivo conditions, it is mainly linear, similar to silicone. We therefore performed our tests at low strains similar to strain variations found in small vessels such as coronary and cerebral arteries [46]. Schneider et al. reported a linear and constant elastic modulus in the range of low strain (0–45%) using continuous data sampling. In comparison, we showed that silicone was slightly viscoelastic within a similar range (0–28%), suggesting that the Young's modulus is dependent of the strain range considered [40].

Another important factor to consider is viscoelasticity as observed during preconditioning cycles and the effect of strain rate. Kim et al. have found that Sylgard 184^®^ displays a different first loading curve as compared to following loading curves, hence viscous energy can be dissipated throughout the material, with this effect being more significant with softer elastomeric materials [43]. This highlights the importance of performing preconditioning cycles as we have done. Khanafer et al. have examined the effect of strain rate (5 and 500 mm/s) on the tensile properties of silicone (curing at 65 °C for 12 h) [17] and found that the Young’s modulus increases with increased strain rate, particularly for softer materials. We have found a slight but not significant increase in stiffness with increased strain rate. This is also in accordance with the results obtained by Colombo et al. [30] who tested strain rates of 0.5, 1.0 and 1.5 mm/s (curing at 120 °C for 1 h). The different results regarding the effect of strain rate are likely due to the relative range of the strain rates tested as well as the experimental procedure and the varying curing conditions between research groups (curing temperature, curing time, base to curing agent ratio).

### Hydrophilization

As stated before, material surface properties can change cell response. Contact angle is a measure of the hydrophobicity of a material and therefore reflects its capacity to interact with aqueous solutions. After curing, silicone is hydrophobic with a contact angle of approximately 108° as reported by others [47, 48]. This compares well with our results as single measures for the different conditions vary from 108.1° to 118.6°. Hydrophobicity prevents aqueous solvents to infiltrate and swell the material. As studies have shown that to maximize cell adhesion the contact angles should be between 60° and 80° [49], surface modifications are required to enhance adhesion of extracellular matrices and to allow adequate conformation of the adsorbed proteins [50], with potential effect on cell behavior and function.

Koh et al. [51] have examined the contact angle of water on silicone treated with sulphuric acid and nitric acid mixed with hydrogen peroxide and found that it decreased to about 75° after 75 s of immersion. We have found that, used alone, sulphuric acid reduced the contact angle to about 100°, whereas it decreased it to values around 90° after immersion in water for 7 days. Mata et al. have analyzed surface properties of silicone after exposure to oxygen plasma and chemical immersion in sulphuric acid (96%). They have found that sulphuric acid alters the surface significantly [19]. The contact angle of untreated silicone found was 113°, independently of the composition of the silicone. This group has not found that hydrochloric acid, hydrogen peroxide, water, hexane, toluene, acetone, methanol or isopropanol altered the contact angle significantly. However, they have measured hydrophobicity 24 h after treatment, hence the surface was potentially affected by hydrophobic recovery.

Changes in hydrophobicity appear to be unstable in air, fading away with time, also called air-induced hydrophobic recovery. Lee et al. [49] found that oxidized surfaces recovered their original contact angle when left in air for about a week, similar to the results we have found. McDonald et al. have studied plasma oxidized surface stability in air and found that they were stable for approximately 30 min. Similarly to our results, they have found that keeping the oxidized silicone in contact with a polar liquid, such as water, protects the surface although long term stability is not guaranteed [52]. They have hypothesized that silicone surfaces do not remain hydrophilic while stored in air due to mobile, low-molecular weight monomers migrating from the bulk of the material to the air-surface interface, increasing hydrophobicity. This hypothesis is supported by investigators which suggested that storage under water reduces the rate of hydrophobicity recovery [48]. Similarly, Morra et al. [53] have used oxygen plasma-treated silicone surfaces stored in air and water and observed increases in surface tension of treated samples, however in air, samples returned to a low-surface-tension. Tan et al. [54] also observed that the contact angle receded after exposure to air. The information about storage conditions after hydrophilization is important as cured silicone can be kept in different ways between laboratories, hence introducing potential difference between labs and batches [16].

### Sterilization

Sterilization can change the hydrophobicity of the materials. We have measured contact angle for different sterilization methods (UV exposure, ethanol immersion and boiling) and have not observed significant differences. This agrees with previous results from Mata et al. [19] which did not observe changes in surface porosity or hydrophobicity after sterilization, except for an increase in contact angle after UV sterilization. Waddell et al. [55] have also reported that UV treatment can alter contact angle. We have not found differences in contact angle after UV exposure, however this could be due to the different UV exposure techniques used.

### Coating agents

In order to provide an adequate coverage of cells within scaffolds, different extracellular matrices have been used to cover the silicone surface, such as collagen, mainly type 1, fibronectin and gelatin, but also vitronectin present in the serum used in culture. Covalent and non-covalent protein coating to the silicone elastomer has been performed by different groups and have been demonstrated to alter attachment, spreading and proliferation of different cell types [23, 27, 28, 30]. We have used overnight coating of collagen type 1 and fibronectin alone or in combination at 37 °C and found no advantage with increasing concentration in terms of cells adhered. Although we have varied concentration, time of coating (varying to minutes to overnight) and temperature have not been studied. Similar to our technique, Tzvetkova et al. [56] have treated silicone surface with fibronectin for 1 h at room temperature with 3.5 mg/cm^2^ which would translate to a high concentration of 43.75 mg/mL in our scaffolds. Colombo et al. [30] have tested a fibronectin concentration of 8 µg/mL for 30 min and found an increased number of adhered cells compared to untreated surfaces. Although these conditions were found to be suitable for cell culture, coverage was not thoroughly assessed and parameters varied. From our results, we can conclude that the collagen and fibronectin coating concentrations appear to have no significant effect on final cell coverage in the ranges of 5–10 and 10–40 µg/mL respectively. Our results are in agreement with Budd et al. [57] which studied the effect of fibronectin concentration and incubation time on the attachment of endothelial cells to PTFE grafts. They have showed that after roughly 30 min of coating with a fibronectin concentration of 10 µg/mL cell attachment does not vary significantly. Others have also studied fibronectin attachment to different substrates [57–61]. As a higher concentration and a longer time of coating were used without increasing significantly the amount of cells adhered, we believe that a fibronectin concentration of 10 µg/mL is adequate for coating the silicone scaffolds.

### Cell seeding

Several interrelated parameters can influence the adhesion of cells to silicone surfaces. Substrate coating depends as detailed above on surface hydrophobicity as well as the length of coating and substrate concentration used. It is important for cell seeding to have an adequate substrate, as it influences cell adhesion and cell function [62]. Another important parameter to consider is the cell seeding concentration. We have used a seeding technique allowing even coverage of all sides of the tubular scaffolds and tested different seeding densities. Higher seeding densities should result in greater numbers of cells present after a reduced time, hence the formation of a monolayer in the endothelial cell context. We have used higher cell seeding concentration in the in vitro tubular scaffolds than the one used in conventional in vitro techniques as cells have a reduced time of contact with the surface. Indeed, the coverage of the sides of the scaffolds require that a rotation (8 rpm) be imposed, ensuring uniform proliferation within the construct but reduced time of contact. The seeding concentrations used are equivalent to seeding densities of 2.0 × 10^4^, 4.0 × 10^4^ and 8.0 × 10^4^ cells/cm^2^. Usual 100% confluency level in tissue culture flasks, 1.0 × 10^4^ cells/cm^2^, is below these concentrations to consider the potential increased number of unadhered cells in the scaffolds under rotation. Uniform cell coverage was obtained at higher seeding cell concentrations as observed using microscopy. The amount of cells recovered as quantified using the Hoescht stain shows a trend for a relationship between higher cell seeding density and adhered in the constructs, as there is a large error when counting cells using this method. Van der Zijpp examined the effect of seeding density on fibronectin coated (50 µg/mL in culture medium by adsorption at 37 °C for 1 h, PBS washing prior to seeding) standard untreated and plasma treated polystyrene (corona discharge or gas-plasma). Similar to us, they have found that higher seeding densities resulted more quickly in a confluent endothelial layer, which was reached at a density of about 4 × 10^4^ cells/cm^2^ (initial seeding densities of 5 × 10^3^–2 × 10^4^ cells/cm^2^) [60]. Our resulting cell densities (up to 2.8 × 10^4^ cells/cm^2^) were lower than Van der Zijpp despite a higher cell seeding concentration of 8.0 × 10^4^ cells/cm^2^. These differences can be attributed to the different cell line used and the more complex geometry of our scaffolds which could prevent optimal cell adhesion as compared to flat surfaces. According to our results, the cell seeding concentration had a more pronounced effect on cell coverage (despite only one condition reached significant difference, but clearly showing a trend for other substrate coating) as opposed to the effect of changing the coating substrate type and concentration.

## Conclusion

The effect on the mechanical and surface properties of the manufacturing and processing techniques commonly used to produce in vitro scaffolds for endothelial mechanobiological studies described in this work can serve as guidelines, highlighting many considerations to take into account. The principal steps involved in manufacturing, as well as the preparation and use of tubular in vitro silicone scaffolds for mechanobiological studies have been examined in order to understand how they truly impact the material properties, cell behavior and therefore the results. Our observations suggest that despite its viscoelastic nature, the curing temperature dictates the material properties that can further be altered by other treatments such as thermal-related sterilization processes, but not hydrophilization or coating. Hydrophilization with sulphuric acid along with storage in water effectively reduced the hydrophobicity of the material, promoting protein coating and cell adhesion. Sterilization techniques have no effect on surface nor mechanical properties except for silicone cured at lower temperature and sterilized using a method involving heat such as boiling. Regarding cell growth in the tubular scaffolds, the number of cells recovered from the scaffolds tends to increase with higher cell seeding concentrations but not as a function of the type or concentration of the coated substrate. Although we have not found that the different substrate concentration tested modified the number of cells adhered, it is possible that the concentration used was higher than the limiting quantity needed. Overall, this work demonstrates the necessity of better characterization of the surface and mechanical properties of the scaffolds and of the procedures used to modify the scaffolds prior to cell culture to ensure reliable and reproducible endothelial mechanobiological studies. As the mechanical and surface properties of silicone scaffolds are characterized, future studies should aim at matching the Young’s modulus to physiological tissue samples to produce more realistic scaffolds and determining the quality and quantity of proteins adhered after coating, as well as their effect on cell function.

## Additional file

**Additional file 1.** Effect of storage conditions (water or air). Data normalized to the mean Young’s modulus of the reference samples (produced in the same batch, tested prior to the storage) at each temperature (n = 3) (Two-way ANOVA, no effect of the storage condition on the silicone stiffness, significant effect of time (aging) (***p < 0.0001), significant effect between reference and samples stored for 7 days).
