# Supplementary figures and images for: Effect of manufacturing and experimental conditions on the mechanical and surface properties of silicone elastomer scaffolds used in endothelial mechanobiological studies
Source: Biomed Eng Online. 2017 Jul 14;16:90. doi: 10.1186/s12938-017-0380-5 (PMC5513328; doi:10.1186/s12938-017-0380-5)

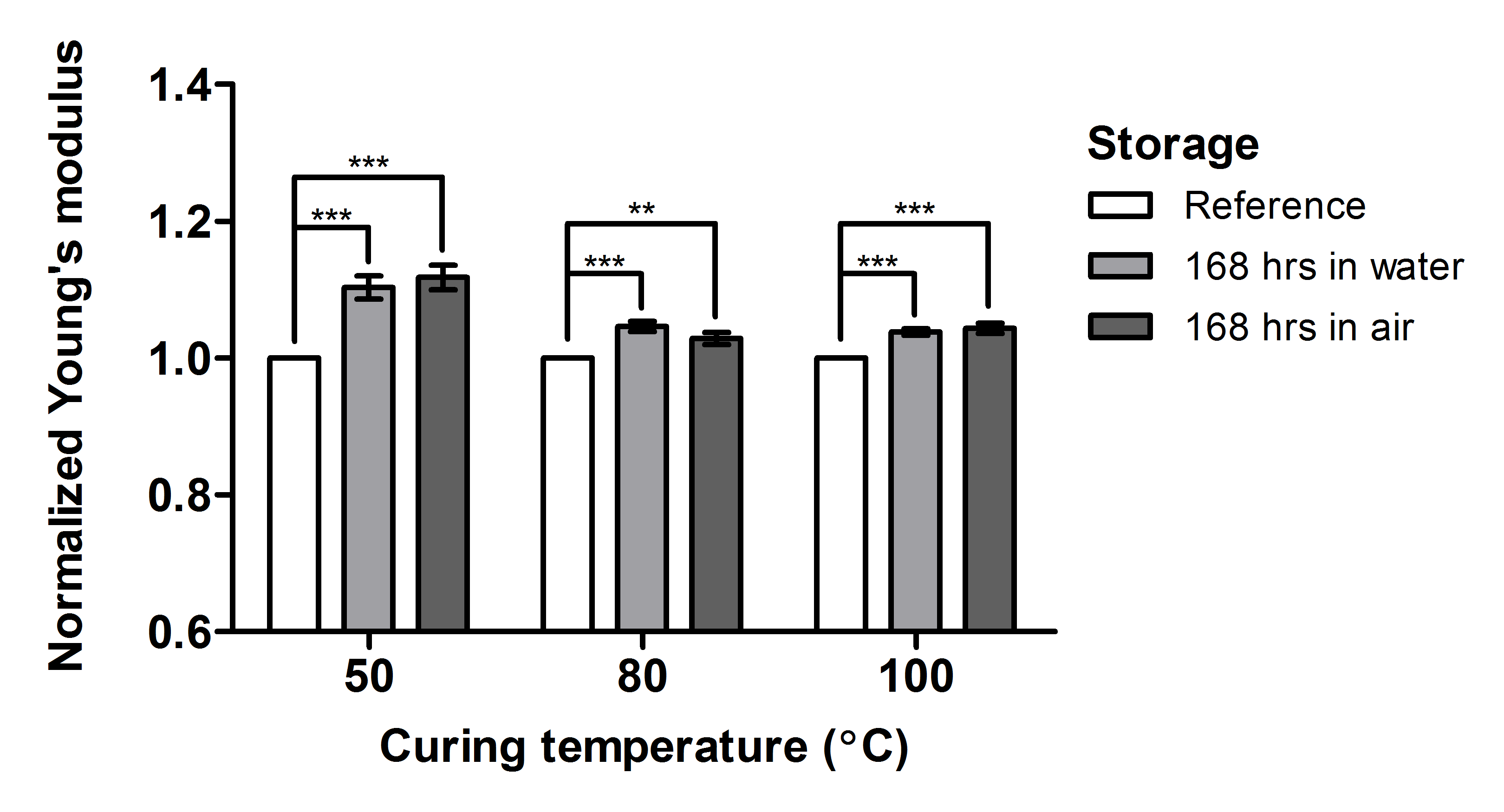

Supplement: Supplementary file 1 — Additional file 1. Effect of storage conditions (water or air). Data normalized to the mean Young’s modulus of the reference samples (produced in the same batch, tested prior to the storage) at each temperature (n = 3) (Two-way ANOVA, no effect of the storage condition on the silicone stiffness, significant effect of time (aging) (***p < 0.0001), significant effect between reference and samples stored for 7 days). [file 12938_2017_380_MOESM1_ESM.tif]
